# P170: Evaluation of knowledge and practices of hospital waste management in Nigeria: implications for the control of healthcare associated infections

**DOI:** 10.1186/2047-2994-2-S1-P170

**Published:** 2013-06-20

**Authors:** E Okechukwu, A Onyenwenyi

**Affiliations:** 1 Centre for Health Research & Development, Action Family Foundation, Nigeria; 2 Institute of Child Health & Primary Care, Lagos University Teaching Hospital, Lagos, Nigeria

## Objectives

The medical waste management is a recognized public health problem, since it exposes healthcare workers, patients and the environment to infection, injury and contamination.

In the era of HIV / AIDS, hepatitis and other epidemics, local data are required to implement policies for prevention and control measures.

## Methods

To assess the knowledge and practices of medical waste management (GDM) in health facilities to facilitate the design activities GDM capacity building to improve the safety of care.

## Results

Internationally validated instruments were used to obtain data from 32 health facilities in four states on the development of key messages, advocacy / awareness sessions. 56 health workers and educational institutions, local authorities were concerned.

After 6 months, capacity building workshops have been made to improve the immediate impact of the project messages on the dangerous practices of GDM prevalent in 97% of schools. A self-administered post-intervention questionnaire was used to compare the pre-test scores.

## Conclusion

In 28 workshops, staff and students 2100 16 educational institutions and health care organizations and 59 civil society have been affected by training on GDM. The post-intervention evaluation showed an improvement of 63% on the issues of knowledge and practice.

Conclusions: This study provides a framework for evidence-based integration of GDM in developing countries to prevent nosocomial infections, promote patient safety and to ensure the sustainability of the healthcare environment.

## Disclosure of interest

None declared

